# Correction: Oncogenic extracellular HSP70 disrupts the gap-junctional coupling between capillary cells

**DOI:** 10.18632/oncotarget.28028

**Published:** 2021-07-20

**Authors:** Dominique Thuringer, Kevin Berthenet, Laurent Cronier, Gaetan Jego, Eric Solary, Carmen Garrido

**Affiliations:** ^1^ INSERM, U866, Faculty of Medecine, Dijon, France; ^2^ CNRS ERL7368, STIM Lab, University of Poitiers, Poitiers, France; ^3^ University of Burgundy, Dijon, France; ^4^ INSERM, U1009, Institut Gustave Roussy, Villejuif, France; ^5^ CGFL, BP77980 21000 Dijon, France

**This article has been corrected:** Due to errors during figure assembly, the ‘calcien’ and ‘phase’ micrograph image panels in Figure 7D are accidental duplicates of the micrograph image panels in row 2, columns 1 and 2, in Figure 6F. The corrected Figure 7, obtained using the original data, is shown below. The authors declare that these corrections do not change the results or conclusions of this paper.

Original article: Oncotarget. 2015; 6:10267–10283. 10267-10283. https://doi.org/10.18632/oncotarget.3522

**Figure 7:**
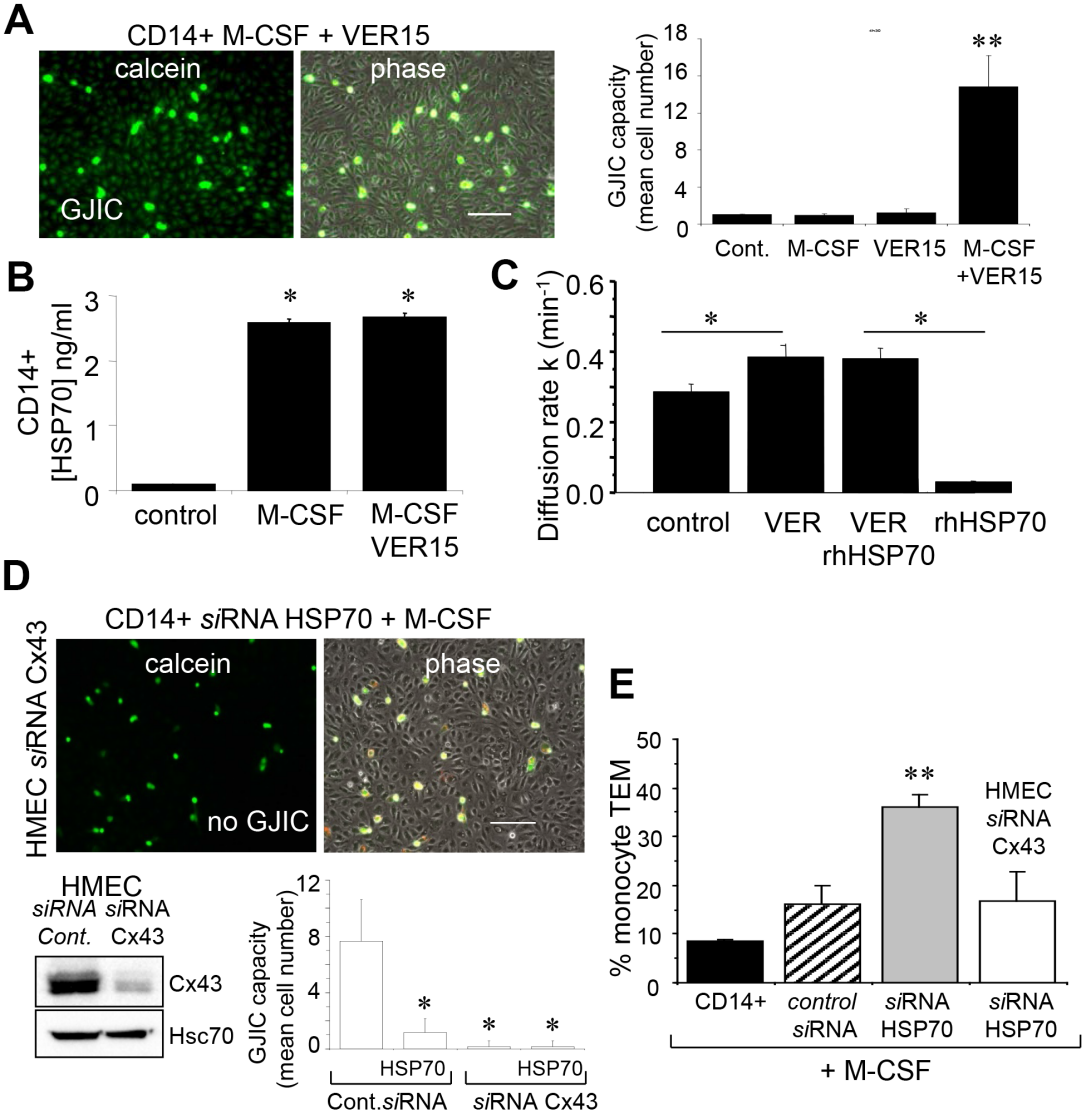
The endothelial Cx43 expression is required for the transendothelial migration of monocytes. (**A**) The adenosinederived inhibitor of HSP70, VER155008 (10 μM) favors the establishment of GJIC between monocytes (M-CSF stimulated) and HMEC within 3 h. Phase-contrast microphotographs are representative of 4 experiments. Bar 100 μm. Right, histogram represents the total cell number of HMEC receiving dye (calcein) per monocyte (mean ±SD, n=3; ^**^P-values <0.01 vs control). (**B**) VER155008 does not inhibit the HSP70 release by M-CSF-stimulated monocytes for 12 h (dosed by ELISA; mean ± SD, n=4; ^*^P-values <0.05 vs control). (**C**) VER155008 antagonizes the blocking effect of rhHSP70 on GJIC between HMEC (gap-FRAP analysis). Histogram shows the constant k measured for the coupled cells after 1 h of cell treatment (mean ± SD, n=4; ^*^P-values<0.05 vs control). (**D**) Effects of the endothelial Cx43 knockdown on the GJIC coupling between HMEC and HSP70 depleted monocytes. Cultured HMEC and monocytes were transfected respectively with siRNA Cx43 (HMEC) and siRNA HSP70 (monocytes) or control siRNA, 48h prior to various analysis. Insert is representative western blot showing the specific depletion of Cx43 in HMEC. Transfected monocytes (donors), stimulated overnight with M-CSF, were pre-loaded with calcein and DiL-C18 before to be plated. Microphotographs of monocytes in contact with transfected HMEC (receivers) after 3 h of culture (representative of 6 experiments; Bar 100 μm). Histogram represents the mean cell number of neighboring HMEC receiving dye (calcein) per monocyte (mean ±SD; n=50 labeled monocytes examined; n=3). (**E**) Effects of the endothelial Cx43 knockdown and released HSP70 on the transendothelial migration of monocytes. Control or transfected HMEC monolayers grown on Transwells were kept in FCSfree conditions overnight. Control or transfected monocytes (3x105 ) stimulated overnight by M-CSF, were labeled with phycoerythrinconjugated anti-CD14+ before to be added into the wells. Cells migrating through the endothelial layers were counted (after 3 h). Data are percentage of total applied monocytes counted by flow cytometry (mean ± SD; n=5).

